# Supplementary material for: Sensory perception of dead conspecifics induces aversive cues and modulates lifespan through serotonin in Drosophila
Source: Nat Commun. 2019 May 30;10:2365. doi: 10.1038/s41467-019-10285-y (PMC6542802; doi:10.1038/s41467-019-10285-y)
Supplement: Supplementary file 3 — Reporting Summary [file 41467_2019_10285_MOESM3_ESM.pdf]

nature research

Corresponding author(s): Scott D. Pletcher

Last updated by author(s): 4/9/2019

Reporting Summary

Nature Research wishes to improve the reproducibility of the work that we publish. This form provides structure for consistency and transparency in reporting. For further information on Nature Research policies, see Authors & Referees and the Editorial Policy Checklist.

Statistics

For all statistical analyses, confirm that the following items are present in the figure legend, table legend, main text, or Methods section.

n/a	Confirmed	
☐	☒	The exact sample size (*n*) for each experimental group/condition, given as a discrete number and unit of measurement
☐	☒	A statement on whether measurements were taken from distinct samples or whether the same sample was measured repeatedly
☐	☒	The statistical test(s) used AND whether they are one- or two-sided<br>*Only common tests should be described solely by name; describe more complex techniques in the Methods section.*
☐	☒	A description of all covariates tested
☐	☒	A description of any assumptions or corrections, such as tests of normality and adjustment for multiple comparisons
☐	☒	A full description of the statistical parameters including central tendency (e.g. means) or other basic estimates (e.g. regression coefficient) AND variation (e.g. standard deviation) or associated estimates of uncertainty (e.g. confidence intervals)
☐	☒	For null hypothesis testing, the test statistic (e.g. *F*, *t*, *r*) with confidence intervals, effect sizes, degrees of freedom and *P* value noted<br>*Give P values as exact values whenever suitable.*
☒	☐	For Bayesian analysis, information on the choice of priors and Markov chain Monte Carlo settings
☒	☐	For hierarchical and complex designs, identification of the appropriate level for tests and full reporting of outcomes
☒	☐	Estimates of effect sizes (e.g. Cohen's *d*, Pearson's *r*), indicating how they were calculated

Our web collection on statistics for biologists contains articles on many of the points above.

Software and code

Policy information about availability of computer code

Data collection	All flies for all experiments were randomized to treatment vials using our DLife survival analysis software or by generating random numbers in the statistical package R (e.g., runif) and then using those random numbers to assign animals and genotypes to treatments and vials.<br><br>In addition to randomization, our survival analysis software allows for blind collection of lifespan data, thus ensuring against bias during data collection.<br><br>All behavioral assays were randomized with respect to treatment and genotype, and all data collection was completed with the experimenter blind to these factors.
Data analysis	For all preference assays, P-values comparing the Preference Index among treatments was obtained using a randomization procedure and the statistical software R. Briefly, the null distribution of no difference among treatments was obtained by randomizing individual preference indices obtained from groups of 20 flies among all measures (maintaining block structure when appropriate) and 100,000 t-statistics (or F statistics for multiple comparisons). P-values (one-sided or two-sided as appropriate) were determined by computing the fraction of null values that were equal or more extreme to the observed t-statistic (or F-statistic). Mean preference values were plotted and weighted by the number of choosing flies in each trial, with the error bars representing the standard error of the mean. Experiment-wise error rates for experiments comparing three or more treatments were protected by presentation of treatment P-value from non-parametric, randomization ANOVA, which are reported in the Figure Legends when appropriate. For lifespan and starvation assays, we employed survival analysis. Unless otherwise indicated, group- and pairwise-comparisons among survivorship curves (both lifespan and starvation) were performed using the DLife computer software and the statistical software R. P-values were obtained using log-rank analysis (select pairwise comparisons and group comparisons or interaction studies) as noted. Interaction P-values were calculated using Cox-Regression when the survival data satisfied the assumption of proportional hazards. In other cases (as noted in the figure legends), we used ANOVA to calculate P-values for the interaction term for age at death. For all box plots, the box represents Standard Error of the Mean (SEM, centered on the mean), and whiskers represent 10%/90%. For CO_2, TAG, and negative geotaxis measures, P-values were obtained by standard two-sided t-test after verifying normality and equality of variances.

All R code that was used in these analyses is available from the authors (spletch@umich.edu).

For manuscripts utilizing custom algorithms or software that are central to the research but not yet described in published literature, software must be made available to editors/reviewers. We strongly encourage code deposition in a community repository (e.g. GitHub). See the Nature Research guidelines for submitting code & software for further information.

Data

Policy information about availability of data

All manuscripts must include a data availability statement. This statement should provide the following information, where applicable:
- Accession codes, unique identifiers, or web links for publicly available datasets
- A list of figures that have associated raw data
- A description of any restrictions on data availability

Metabolomics data and analyses are provided as Supplementary File 1. All additional data and analysis scripts that support the findings of this study are available from the corresponding author on request.

Field-specific reporting

Please select the one below that is the best fit for your research. If you are not sure, read the appropriate sections before making your selection.

☒ Life sciences ☐ Behavioural & social sciences ☐ Ecological, evolutionary & environmental sciences

For a reference copy of the document with all sections, see nature.com/documents/nr-reporting-summary-flat.pdf

Life sciences study design

All studies must disclose on these points even when the disclosure is negative.

Sample size	Sample size was determined based on extensive preliminary studies to ensure statistical power greater than 0.9.
Data exclusions	Data were not excluded.
Replication	Replication was extensive throughout the manuscript, as detailed in the Methods and Figure legends. Key experimental results were repeated independently at least twice.
Randomization	All experiments were subject to randomization of genotype and treatment.
Blinding	As described above, data from all experiments were collected with the experimenter blind to treatment and genotype.

Reporting for specific materials, systems and methods

We require information from authors about some types of materials, experimental systems and methods used in many studies. Here, indicate whether each material, system or method listed is relevant to your study. If you are not sure if a list item applies to your research, read the appropriate section before selecting a response.

Materials & experimental systems

n/a	Involved in the study	
☒	☐	Antibodies
☒	☐	Eukaryotic cell lines
☒	☐	Palaeontology
☐	☒	Animals and other organisms
☒	☐	Human research participants
☒	☐	Clinical data

Methods

n/a	Involved in the study	
☒	☐	ChIP-seq
☒	☐	Flow cytometry
☒	☐	MRI-based neuroimaging

Animals and other organisms

Policy information about studies involving animals; ARRIVE guidelines recommended for reporting animal research

Laboratory animals	The laboratory stocks w1118, Canton-S, UAS-dTrpA1, norpA, and Ir76b-/- [BL51309] Drosophila lines were obtained from the Bloomington Stock Center. PoxnΔM22-B5ΔXB and PoxnFull1 were provided by J. Alcedo 49. Orco2 mutant flies were a generous gift from L. Vosshall 50. Gr63a1 mutant flies were a gift from A. Ray. 5-HT2APL00052 mutant and 5-HT2A-GAL4 (3299-GAL4) flies were graciously provided by H. Dierick. Ir8a-/-/Ir25a-/- mutant flies were kindly provided by R. Benton. Three species of Drosophila (D. simulans, D. erecta and D. virilis) were generously provided by P. Wittkopp. All of these strains were maintained on standard food at 25°C and 60% relative humidity in a 12:12 h light:dark cycle.

Wild animals	*Provide details on animals observed in or captured in the field; report species, sex and age where possible. Describe how animals were caught and transported and what happened to captive animals after the study (if killed, explain why and describe method; if released, say where and when) OR state that the study did not involve wild animals.*
Field-collected samples	*For laboratory work with field-collected samples, describe all relevant parameters such as housing, maintenance, temperature, photoperiod and end-of-experiment protocol OR state that the study did not involve samples collected from the field.*
Ethics oversight	*Identify the organization(s) that approved or provided guidance on the study protocol, OR state that no ethical approval or guidance was required and explain why not.*

Note that full information on the approval of the study protocol must also be provided in the manuscript.
